# Supplementary material for: In Vivo Evaluation of PCL Vascular Grafts Implanted in Rat Abdominal Aorta
Source: Polymers (Basel). 2022 Aug 15;14(16):3313. doi: 10.3390/polym14163313 (PMC9412484; doi:10.3390/polym14163313)
Supplement: Supplementary file 1 [file polymers-14-03313-s001.zip › Supplementary 1 tensile tests.pdf]

Supplementary figure 1. SEM images and stress/strain curves of non-sterilized (A), sterlilzed under 37 °C (B) and 60 °C (C) PCL scaffolds. Bar 20 μm.

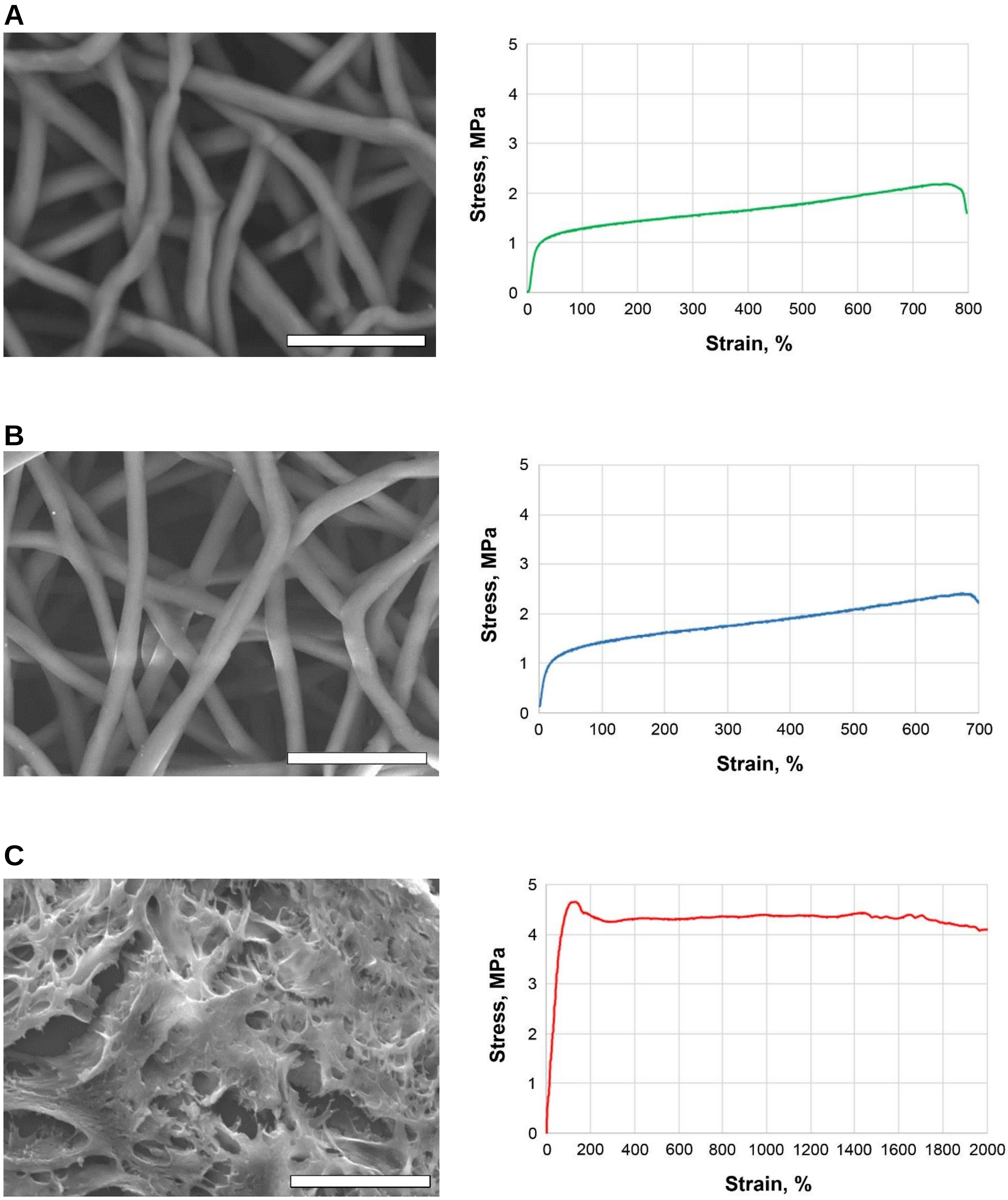

For mechanical tests, 10 mm (length) x 9 mm (width) rectangular fragments were cut out of obtained scaffolds (n = 27), placed in the grips of the device, and stretched longitudinally until failure at an extension rate of 10 mm/min using an ESM 303 L tester (Mark-10 Corporation, USA) and a computer-linked force gauge (0–100 N). All samples were tested dry, without any preconditioning.

Strength was evaluated as failure stress (σ, MPa) (equation 1):

$$\sigma = \frac{Fmax}{S}$$

where σ is the failure stress, Fmax is the peak load, and S is the cross-sectional area of the sample.
Failure strain (ΔE, %) was calculated using equation 2:

$$\Delta E = \frac{Lmax - L0}{L0} \times 100$$

where ΔE is the failure strain, L0 is the initial sample length (mm) equal to the distance between the grips, and Lmax is the maximal deformation (mm).

Results were plotted as stress/strain curves.
