# Supplementary material for: In Vivo Evaluation of PCL Vascular Grafts Implanted in Rat Abdominal Aorta
Source: Polymers (Basel). 2022 Aug 15;14(16):3313. doi: 10.3390/polym14163313 (PMC9412484; doi:10.3390/polym14163313)
Supplement: Supplementary file 1 [file polymers-14-03313-s001.zip › Supplementary 4 Element analysis.pdf]

Supplementary figure 4. SEM-EDS elemental mapping of dried explanted graft at 60 days after implantation.

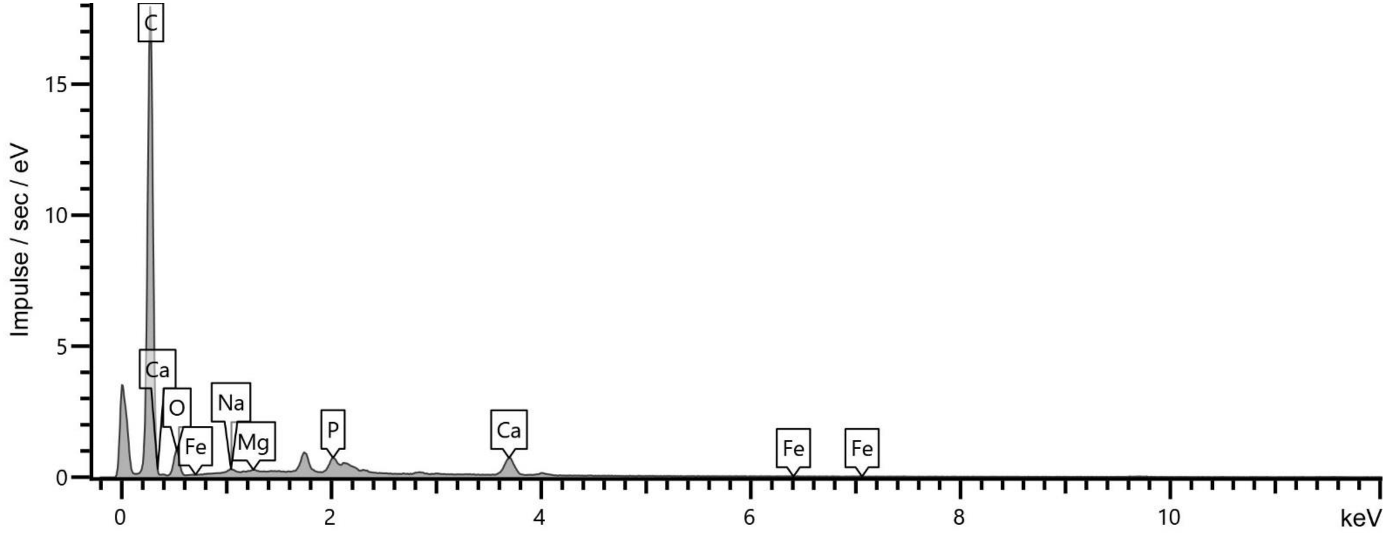

Element	Weight %	Atomic %
C	85.38	90.09
O	10.78	8.54
Ca	2.60	0.82
Fe	0.05	0.01
Na	0.26	0.14
P	0.83	0.34
Mg	0.11	0.06
Total	100	100

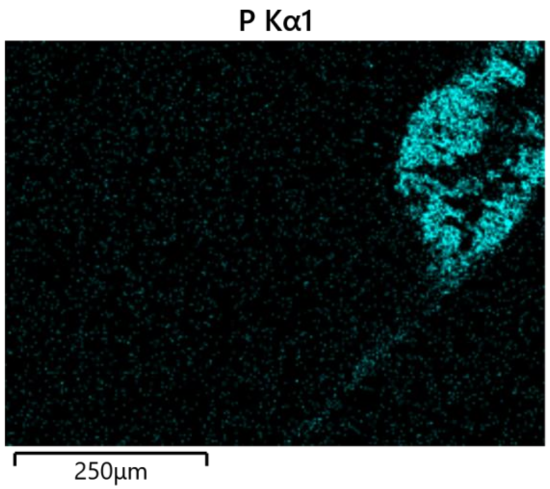

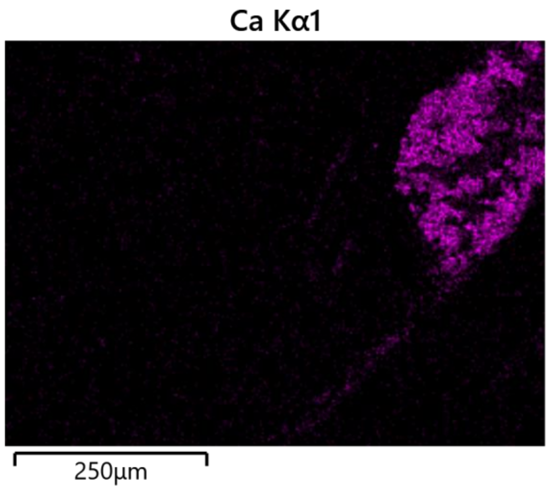

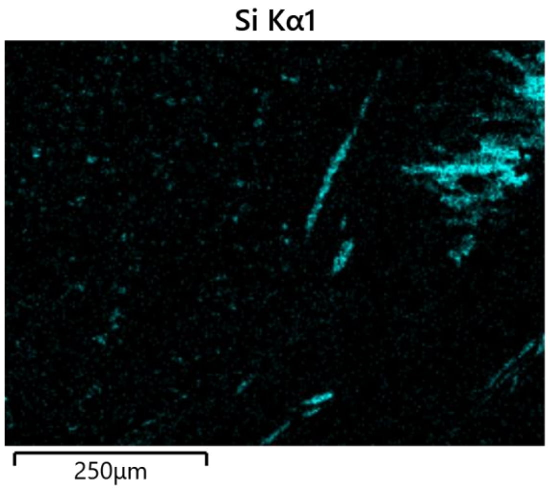

OXFORD INSTRUMENTS
The Business of Science®

C Kα1_2

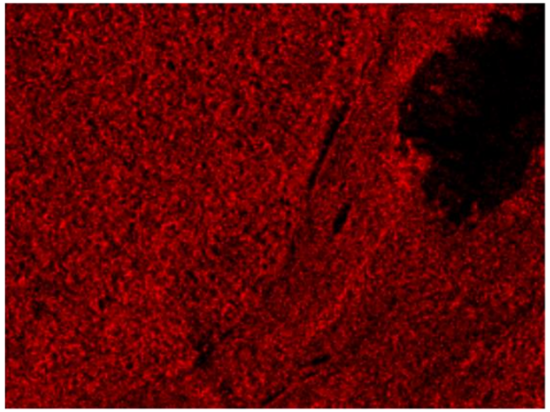

250µm

O Kα1

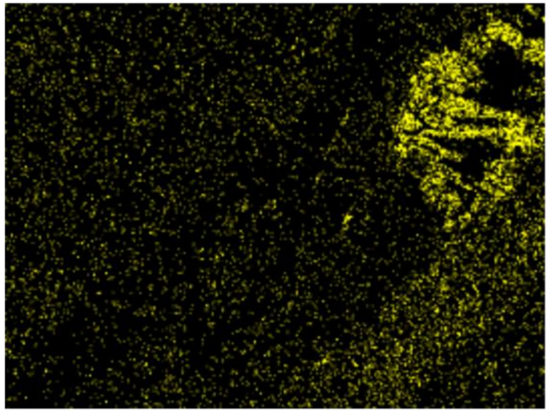

250µm

Na Kα1_2

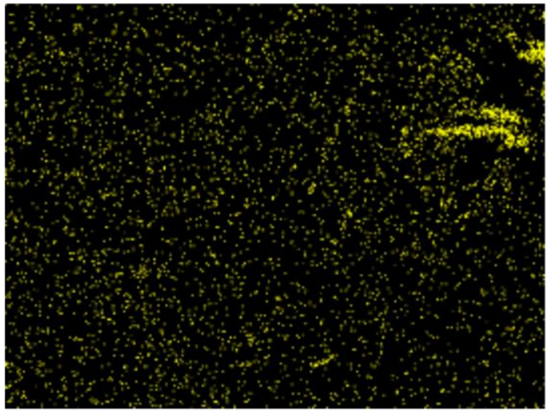

250µm

N Kα1_2

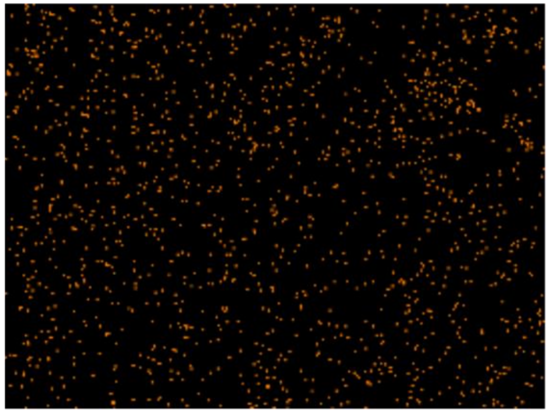

250µm

OXFORD INSTRUMENTS
The Business of Science®
