# Supplementary figures and images for: In Vivo Evaluation of PCL Vascular Grafts Implanted in Rat Abdominal Aorta
Source: Polymers (Basel). 2022 Aug 15;14(16):3313. doi: 10.3390/polym14163313 (PMC9412484; doi:10.3390/polym14163313)

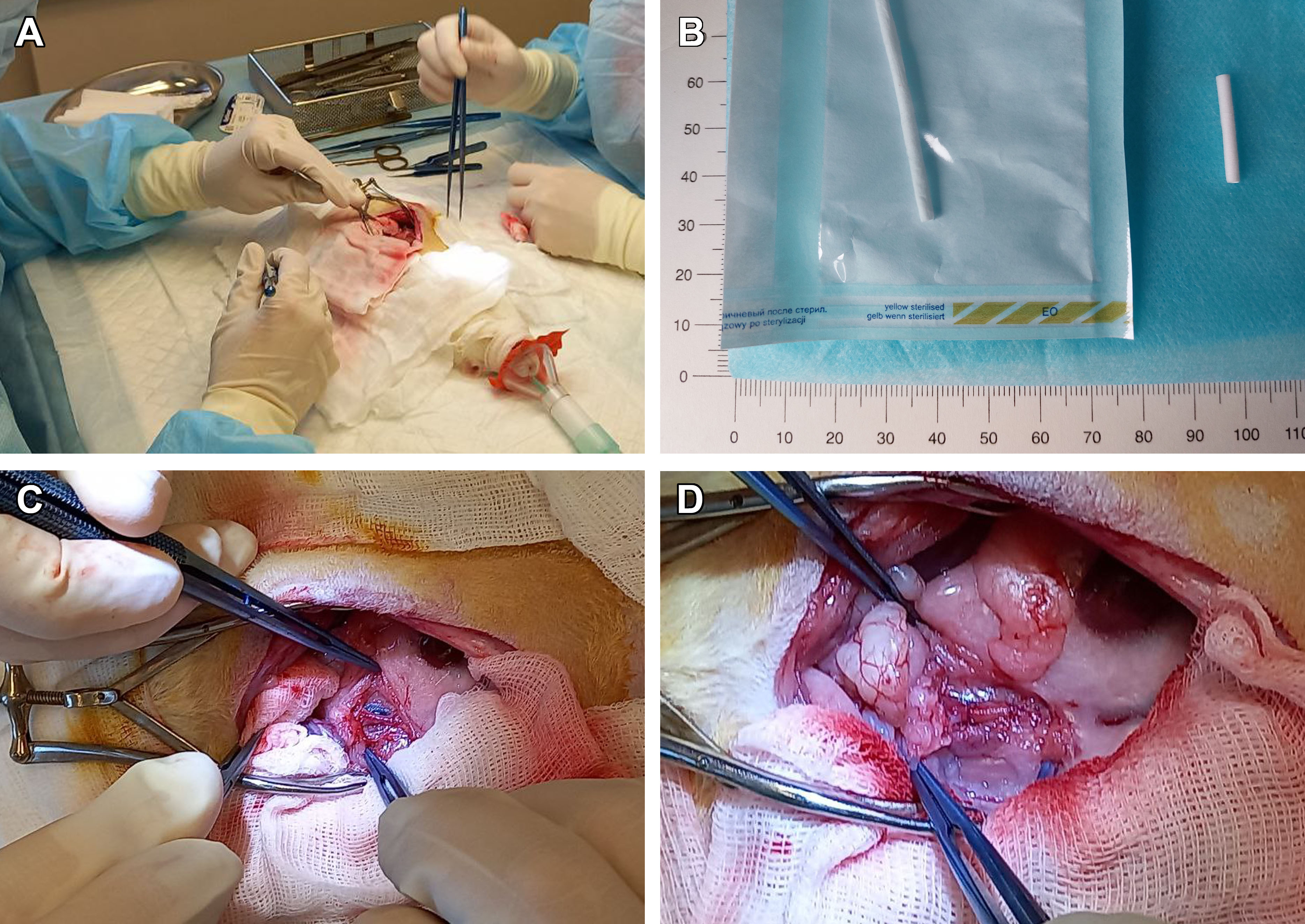

Supplement: Supplementary file 1 [file polymers-14-03313-s001.zip › Supplementary 2 operation.jpg]

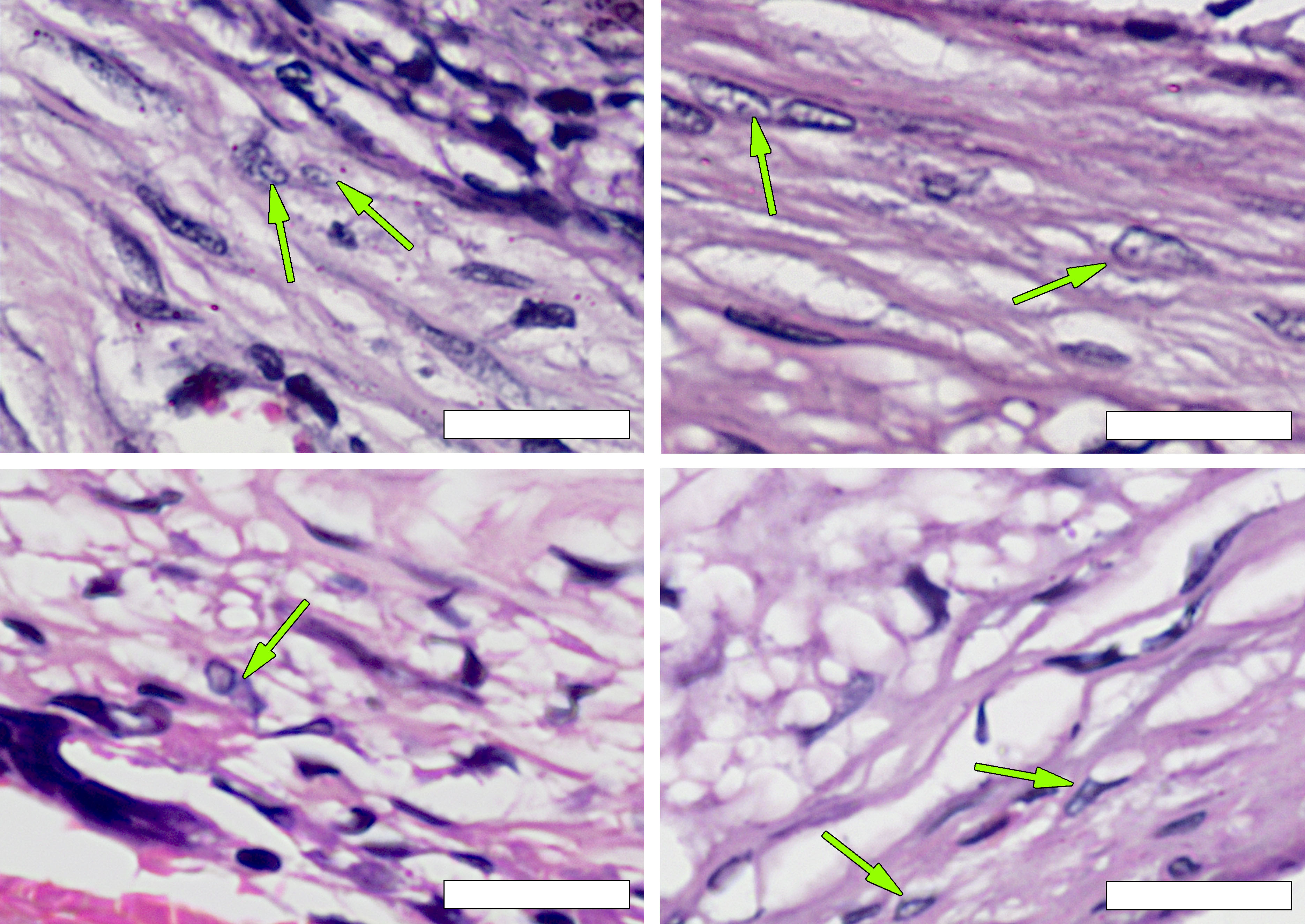

Supplement: Supplementary file 1 [file polymers-14-03313-s001.zip › Supplementary 3 chondroid metaplasia.jpg]
